# Correspondence

**Published:** 1860-06

**Authors:** 


### Correspondence.

Messrs. Editors :—A few days since, I came across another of these peripatetic teeth assassins, in a back street, near what is here known as the "Barbary coast," a locality exceedingly dangerous to financial navigation in consequence of the innumerable shoals and land sharks there abounding. He was conspicuously "topped off" with a green velvet cap, which was bespangled with a fabulous number of defunct molars sewed on in regular rows and all centering at the tassel at the apex, presenting, as you may suppose, a very striking and picturesque appearance. In his hand he held a substance of a pink color similar in appearance to a cake of toilet soap, and under his left arm the head of about a ten year old girl (on another occasion the head of a boy, and to which heads I wish it understood, there were bodies attached.) He was applying a portion of this substance to the incisors of the victim, expatiating meanwhile, to a curious crowd, on the virtues and transcendent qualities of his tooth soap in cleansing and whitening the teeth. He made no sales while I was present and I trust may make none, and thus be driven to some useful employment.

*Sponge or Crystal Tin*, which was noticed in the Dental Review for May, will not, I fear, result in any good. From some experiments made, I have discovered that the moment the crystals are removed from the solution, and washed free from acid, their surfaces become oxydized. While in the acid the surfaces are kept bright which secures strong adhesion, but the moment they become clouded or dimmed, as they do almost instantly in air or water, the adhesive quality is lost, and on pressure they fall into powder.

A friend suggests in this connection that a chemically pure tin foil, or at least tin entirely free from lead, is a desideratum in the profession.

*Osteoplasty,—os—artificial,* etc., are now assuming some importance.

A few days since a gentleman in the profession exhibited to me an upper set of teeth, of a block in one piece, mounted upon a silver plate by a composition resembling "hard rubber" the only attachment being this intervening substance. Now as it is well known that in consequence of the sulphur in the rubber acting upon silver, that the two cannot be combined, I was led to reflect as to the nature of this substance when the recollection of some experiments, made in this direction nearly four years ago with the oxyde of zinc, came into my mind, another secret was out.

These experiments, and which I desire to refer to here, were made with a freshly calcined oxyde of zinc and a highly concentrated chloride of zinc; and from the very hard substance produced by their combination (to which any appropriate color may be given by the addition of metallic pigments,) it was hoped that a plastic material had been discovered which could be made valuable in filling and mounting teeth. Accordingly a number of teeth were filled, and a piece of mechanical work, very creditable in appearance, was gotten up, in which single teeth were firmly and neatly mounted upon silver, presenting the general appearance of continuous gum work, and the only apparent objection to the material, at that time, was its *pungency* in the mouth.

The experiments however were discontinued, on its being demonstrated that the plugs were gradually dissolved—*that they would not bear the test of time.* Other substances, I suppose, might have been combined with the zinc to give it greater density and thus make it less liable to dissolve or disintegrate, or wear away in mastication, as well as reduce its unpleasant pungency; all of which, it is to be hoped, has been done by the gentlemen introducing these substances at this time, otherwise—and even with the additions, I fear—disappointment awaits all who have employed it in their practice. For other than temporary work I should be afraid to trust it, and I do hope therefore that some caution will be used in its employment as a filling.

*A review* of Tomes and Taft's recent works has just appeared in the "North American Medico-Chirurgical Review" for May. The article occupies some twelve pages, over four of which are devoted to the books in question, and the balance of eight pages to a history of the progress of dentistry in the United States. In regard to the first named work, the reviewer regrets that so large a portion of the book should be devoted to teething, which, he thinks, foreign to the subject, but with the further exception of the discussion of "dental tissues," (which it is thought does not properly belong in a work bearing its title,) the reviewer commends the balance of the book without reservation and says "to the remainder of the work we could cheerfully annex our sign manual," etc. Being so well pleased with the book he expresses a "regret that Mr. Tomes did not furnish us that which we so much need, a *complete* system of dental surgery," and further compliments it as "a model of composition at once pure, chaste and classical," and that it is "one of the most readable scientific books in the English language." As to Taft's work, he is not so complimentary, which is owing, he tells us, to "certain defects," "both of a scientific and literary character," which defects he notices with regret, because it is an American publication, but attributes these objectionable points to "a want of caution and consideration" and "not through ignorance of the author" and hinting at "precipitate" preparation and asserting its premature appearance.

In support of his charge of defects he cites the position taken by the author, of the inability of the teeth to undergo sudden transitions of temperature; questions whether there is such a condition as "inflammation of dentine," denies that the hardening of amalgam fillings "is caused mainly by evaporation of the mercury," with some two or three others of minor importance. Some of which defects, as will be seen, are of a controversial character.

He speaks kindly of the *illustrations* in the fourth chapter of the work—on instruments for filling and their manufacture,—and winds up with the expression of a hope that what has been said " will not be attributed to a spirit of fault-finding" and further says that "book-reviews are calculated to stimulate future authors to greater watchfulness rather than condemn works already written." This is certainly *very* encouraging to future authors. Now, after a careful reading of this review, and noting particularly the very limited number of defects that he has pointed out in the book, and the entire absence of a single word of commendation, I submit to the unprejudiced reader of this review whether there is not evidence of a palpable, manifest indisposition on the part of the reviewer to discover any good in it, and whether a spirit of complaint and fault-finding does not pervade the whole review? Why the book is not even " damned with *faint* praise," for there is not a single sentence that could by any known process of distortion be made to assume an equivocal meaning of a complimentary character; but the author is patted on the back and told that his " character as an operator, and as one anxious for the advancement of the profession, is second to none, and being fully competent to the task he undertook," etc., yet his production is unqualifiedly condemned, from the first to the last page, without the mention of one redeeming feature that could with honor be accepted by the author; and all this based upon some five or six defects only, which are all that has been cited, and which are offered, *and all that are offered*, be it remembered, in justification for such an unkind, unqualified, condemnatory critique.

Of one thing I feel assured, and that is that this review will operate on the profession as an overdose of a violent poison frequently acts upon the human system.

The remainder of the review, amounting to some eight pages, is devoted to a history of dentistry and commences with some reference to its antiquity, and noting its rapid improvement—more particularly mechanically—down to the present day, forming an interesting paper, a portion of which is a grouping together of many facts and statistics that have been published from time to time in the periodical press of the profession; also including some notice of the Dental periodicals with some suggestions to one of them, as to what is *not* proper matter for publication; and a criticism on the life, character and management of another now defunct; and information to the profession as to where they may obtain true, legitimate, nutritious professional pabulum, and which constitutes a first-rate notice of a third; and then closes with the hope that at some future day, not far distant, we "shall be called upon to notice some marked evidence of the continued advance of a science the prosperity and ultimate triumph of which we shall hail with the highest gratification."

Yours, O. U. C.

*Philadelphia, May* 11, 1860.

